# The Role of Collagen Rheology in Human Keratinocyte Differentiation: Implications for Skin Substitute Development

**DOI:** 10.3390/polym17172325

**Published:** 2025-08-28

**Authors:** Mirna Rodríguez-Aguilar, Blanca Segura-Pacheco, Bernardo Campillo-Illanes, María Soledad Córdova-Aguilar, Horacio Merchant-Larios, Sergio Alcalá-Alcalá, Angélica Meneses-Acosta

**Affiliations:** 1 Laboratorio 7, Biotecnología Farmacéutica, Facultad de Farmacia, Universidad Autónoma del Estado de Morelos, Avenida Universidad No. 1001, Colonia Chamilpa, Cuernavaca 62210, Morelos, Mexico; mirna.rodrigueza@uaem.edu.mx; 2 Triovance Holding LLC, San Diego, CA 92119, USA; b.segura@triovance.com; 3 Instituto de Ciencias Físicas, Universidad Nacional Autónoma de México, Avenida Universidad No. 1001, Colonia Chamilpa, Cuernavaca 62210, Morelos, Mexico; bci@icf.unam.mx; 4 Laboratorio de Ingeniería de Proceso, Instituto de Ciencias Aplicadas y Tecnología, Universidad Nacional Autónoma de México, Mexico City 04510, Mexico; marisol.cordova@icat.unam.mx; 5 Departamento Biología Celular y Fisiología, Instituto de Investigaciones Biomédicas, Universidad Nacional Autónoma de México, Circuito Escolar, Edificio A, Mexico City 04510, Mexico; merchant@unam.mx; 6 Laboratorio 1, Tecnología Farmacéutica, Facultad de Farmacia, Universidad Autónoma del Estado de Morelos, Avenida Universidad No. 1001, Colonia Chamilpa, Cuernavaca 62210, Morelos, Mexico

**Keywords:** collagen hydrogels, skin substitutes, tissue engineering, keratinocytes, extracellular matrix, biocompatible materials, cell differentiation

## Abstract

Type I collagen hydrogels are widely employed as scaffolds in tissue engineering due to their biocompatibility and ability to mimic the extracellular matrix (ECM). ECM viscoelasticity plays a critical role in regulating key cellular functions such as adhesion, proliferation, and differentiation. This study evaluates how collagen source and quality influence hydrogel architecture, mechanical properties, and keratinocyte behavior. Hydrogels were prepared at a concentration of 2.3 mg/mL using collagen from Advanced Biomatrix (AB, GLP grade) and Collagen Solutions (CS, GMP grade), and assessed for fibrillogenesis, rheological performance, and their ability to support stratified HaCaT keratinocyte cultures. AB-derived hydrogels exhibited higher porosity but lower mechanical resilience, characterized by a linear viscoelastic region (LVER) of 2.54%. In contrast, CS-derived hydrogels showed reduced porosity, denser fiber networks, and a higher LVER of 9.96%, indicating enhanced strain tolerance. HaCaT cells cultured on AB hydrogels showed diminished proliferation, metabolic activity, stratification, and expression of differentiation markers compared to those on CS hydrogels, which supported a more robust epidermal architecture. These findings highlight the critical role of collagen quality and mechanical characteristics on scaffold performance and epidermal tissue formation, emphasizing the need to optimize biomaterial properties for effective regenerative outcomes.

## 1. Introduction

Regenerative medicine has emerged as a transformative approach to tissue engineering (TE), a discipline focused on restoring or replacing damaged tissues and organs, with particular emphasis on skin regeneration [1]. The increasing demand for alternative therapies to treat a range of skin conditions has driven significant progress in this field [2]. Advances in tissue engineering and biomaterial design have enabled the development of various artificial skin substitutes that offer clinically effective solutions for patients requiring dermal replacement. These innovations have markedly reduced morbidity and mortality associated with chronic wounds and extensive skin damage [3,4].

The successful development of skin substitutes is based on the integration of three fundamental components: *scaffolds* (biopolymers), *growth factors* (cell culture media), and *specialized cells* [5]. Continuous research efforts have led to the discovery of novel materials and fabrication techniques for constructing these complex systems [6]. Three-dimensional (3D) scaffolds play a pivotal role by mimicking the extracellular matrix (ECM), thereby providing a structural and biochemical framework that supports cell adhesion, proliferation, and differentiation [7,8]. Designing an optimal scaffold requires the selection of appropriate biopolymers—primarily ECM-derived proteins such as collagen, hyaluronic acid, fibronectin, and fibrin—which provide diverse microenvironments conducive to tissue development and regeneration [9].

Hydrogels, as 3D scaffolds, have demonstrated exceptional versatility in tissue engineering applications due to their inherent biocompatibility, biodegradability, remodeling capacity, and ability to support essential biological functions [5,10]. Type I collagen is the most widely used natural biopolymer in hydrogel-based scaffolds, owing to its role as the primary structural and functional component of the dermal extracellular matrix (ECM). Type I collagen is a triple-helical protein with a molecular weight of approximately 300 kDa, distinguished by its 67 nm D-periodicity within fibrillar assemblies [11,12]. Under physiological conditions, collagen molecules spontaneously self-assemble into fibrils and form a hydrated gel—composed of over 90% water—that provides both structural integrity and mechanical support within the extracellular matrix [13,14].

Collagen can be isolated from connective tissues such as skin and tendons using either acidic (e.g., HCl) or enzymatic (e.g., pepsin) extraction methods. These processes yield two main forms of collagen: telocollagen, which retains the non-helical telopeptide regions, and atelocollagen, in which these regions have been removed by enzymatic treatment [15]. Beyond its native structure, collagen offers numerous reactive functional groups—primarily free amine and carboxyl moieties—that can be chemically or physically modified to tailor its physicochemical properties. These functional groups allow the formation of crosslinks, enhancing the mechanical stability, degradation resistance, and overall performance of collagen-based scaffolds in tissue engineering [16,17].

The capacity of collagen to emulate the ECM microenvironment arises from the formation of a hydrated network of biomacromolecules that modulates key cellular processes—such as motility, proliferation, differentiation, and apoptosis—through the transmission of biochemical and biophysical signals. These cellular responses are tightly regulated by the viscoelastic properties of the ECM [13,18]. Collagen also exerts biological activity through receptor-mediated signaling, primarily via integrins, which form physical links between the extracellular matrix and the intracellular cytoskeleton [19]. This integrin-mediated mechanotransduction is critical for translating external mechanical signals into intracellular responses. Indeed, the dynamic balance of mechanical forces between cells and their surrounding matrix has been identified as a central determinant of cell morphology, function, and fate [20].

Despite the favorable biological properties of collagen-based scaffolds as a natural biomaterial, several limitations continue to restrict their broader application in tissue engineering. The main factors that negatively influence their performance include the tissue of origin, the extraction and solubilization method, the pH of the solution, the polymerization temperature, the ionic strength, the final collagen concentration, and, in particular, their low mechanical performance [21,22,23].

To overcome these limitations, combinations of collagen with other natural polymers such as polysaccharides (cellulose, chitosan, alginate, hyaluronic acid), fibrin, and gelatin have been explored, offering advantages such as biodegradability, biocompatibility and versatile functionality [24]. Bindi et al. (2023) designed and characterized hydrogels based on collagen, hyaluronic acid, and fibrin, which exhibited viscoelastic properties comparable to native skin, optimized porous architecture and good biocompatibility with human fibroblasts, highlighting their potential for clinical applications [25]. Similarly, Sánchez-Cid et al. (2024) reported improvements in the rheological properties of hydrogels combining collagen with chitosan through controlled crosslinking, which reinforces their applicability in tissue regeneration [26]. Likewise, Ramasamy et al. (2021) developed a full-thickness skin model by incorporating fibroblasts into a type I collagen hydrogel chemically crosslinked with polyethylene glycol four-arm succinimidyl glutarate (PEG-SG), which stabilized interfibril connections, reduced shrinkage, and minimized cell remodeling [27]. These studies collectively highlight how scaffold structural and mechanical factors influence fibrillogenesis, functional integrity and cellular behavior, thereby complicating the standardization of collagen-based scaffolds to achieve reproducible and functional skin constructs in advanced clinical applications [28,29,30].

To minimize complications associated with the standardization of collagen crosslinking methodologies, commercially available collagen products manufactured under Good Manufacturing Practices (GMPs) and Good Laboratory Practices (GLPs) have been adopted. These products undergo rigorous characterization, including evaluation of fibrillar architecture, purity, telopeptide content, polymerization kinetics, and the structural and mechanical properties of the polymerized fibrils [23,31]. This approach offers a cost-effective and reproducible method for generating hydrogels suitable for tissue engineering applications.

Although various studies have investigated the influence of collagen source and mechanical properties on tissue regeneration, the quality of the biomaterial itself is often overlooked. Currently, there are no direct comparisons between collagen products with different production grades specifically aimed at the development of skin substitutes. This study presents a comparative evaluation of two bovine Type I/III collagen products with distinct production standards.

Collagen AB (Advanced Biomatrix), produced under GLP-grade conditions, is designed to ensure the repeatability of experimental results in preclinical research. While its specification lists a composition of 97% Type I collagen and 3% Type III, the certificate of analysis states only that ≥85% is atelocollagen. This product is primarily intended for in vitro research, offering high batch-to-batch consistency [32].

In contrast, Collagen CS (Collagen Solutions), manufactured under GMP standards (ISO 13485 and ISO 22442), adheres to strict guidelines for production, quality control, traceability, and storage, making it suitable for clinical applications. According to its technical documentation, it contains 95% Type I atelocollagen and 5% Type III, ensuring higher purity and biocompatibility. This level of quality is particularly relevant for the development of skin substitutes intended for treating chronic wounds [33].

This study aims to characterize bovine Type I 3D collagen hydrogels to evaluate their capacity to serve as a three-dimensional scaffold for cell sheet formation, to assess whether collagen quality influences the differentiation of human keratinocytes, and to investigate whether the rheological properties of these hydrogels affect cell growth.

## 2. Materials and Methods

### 2.1. Collagen

Collagen hydrogel mixtures were prepared according to the supplier’s instructions, utilizing two commercial sources of Type I collagen. The first source, *Collagen AB*, was PureCol^®^ bovine collagen (Advanced Biomatrix, REF. 5005-100ML, Carlsbad, CA, USA). This product consists of 97% Type I atelocollagen and 3% as Type III collagen, supplied at a concentration of 3 mg/mL pepsin-solubilized in 0.01 N HCl, and designated GLP Grade. The second source, *Collagen CS*, was a highly purified Type I bovine atelocollagen (95%) and 5% as Type III collagen (Collagen Solutions, REF. FS22002, Eden Prairie, MN, USA). This product was also supplied at a concentration of 3mg/ML in 0.01 N HCI and designated GMP Grade. To neutralize the collagen solutions, one part of chilled sterile 10X PBS (pH 7.4; phosphate-buffered saline, Gibco, REF. 14200-075, Grand Island, NY, USA) was gently mixed with eight parts of chilled collagen solution via swirling. The pH of both collagen mixtures was then adjusted to 7.0–7.5 using sterile 0.1 M NaOH. This neutralization process, as proposed by the suppliers, resulted in a final collagen concentration of 2.3 mg/mL for both sources. These concentrations are the result of neutralization and mixtures proposed by suppliers, which were followed in order to maintain a standardized methodology.

### 2.2. HaCaT Cells

Human HaCaT keratinocytes (Creative Bioarray, REF. CSC-C8977H, Shirley, NY, USA) were cultured in Keratinocyte Serum Free Medium (SFM, Gibco, REF. 10724-011, Grand Island, NY, USA), supplemented with 5 ng/mL recombinant human epidermal growth factor (rEGF), 50 µg/mL bovine pituitary extract (BPE) (Gibco, REF. 370000-015, Grand Island, NY, USA), and 0.03 mM calcium chloride (low calcium) at pH 7.2. Cells were maintained at 37 °C in a culture incubator with 5% CO_2_ (Eppendorf New Brunswick S41i, Serial No. 45254, Enfield, CT, USA).

### 2.3. Characterization of Collagen Hydrogels

#### 2.3.1. Collagen Hydrogel Fibrillogenic Kinetics

A turbidimetric assay was used to analyze the polymerization (fibrillogenesis) kinetics of each collagen source. The assay was performed using a spectrophotometer (Thermo Scientific, Multiskan Sky with a cuvette and touch screen, Serial No. 1530-800959C, Waltham, MA, USA) equipped with a Peltier temperature controller set to 37 °C. Absorbance at 310 nm was recorded every 2 min for up to 2 h ( n=9 ). Following the characteristic fibrillogenesis curve described by Antoine et al. (2014) [23], four parameters were extracted from the absorbance profiles: *total change in absorbance* ( ΔAbs ); *polymerization half-time* (t_1/2_) defined as the time taken to reach half of ΔAbs ; *polymerization rate* ( $\frac{dAbs}{dt}$ ), calculated as the slope at (t_1/2_) and *lag time* ( $t_{L}$ ), defined as the intercept on the time axis of the line passing through the point [t_1/2_, ΔAbs ] with slope $\frac{dAbs}{dt}$ (where absorbance equals zero).

#### 2.3.2. Morphological Analysis of Collagen Hydrogels

Morphological analysis was conducted using scanning electron microscopy (SEM). Collagen hydrogels were prepared as described in Section 2.1 (2.3 mg/mL) and subsequently lyophilized in FreeZone 4.5 (LABCON, Kansas City, MO, 64132, USA) at 99 Pa. The condenser temperature was maintained at −50 °C, and sublimation was carried out at room temperature. Images were captured using a JEOL 5900-LV variable pressure scanning electron microscope (JEOL, Peabody, MA, USA) at 10 kV and magnifications of 500× and 3000×. The operating conditions were set to a pressure of 20 Pa and an accelerating voltage of 15 kV. The mean diameters of the nanofibers were determined by measuring 120 individual structures from the SEM images using the ZEN lite 3.1 Blue Edition software (ZEISS Microscopy, Oberkochen, Germany).

#### 2.3.3. Rheology of Collagen Hydrogels

Rheological characterization was performed using a Physica MRC 101 rheometer (Anton Paar Instruments, Graz, Austria), equipped with a 25 mm diameter flat-plate geometry (PP25) and a Peltier heating stage maintained at 37 °C. Collagen AB and CS mixtures (2.3 mg/mL, pH 7.4) were carefully applied to the rheometer stage, and the measuring geometry was set to a 1 mm gap, gently compressing the sample by approximately 15%. To prevent sample dehydration during the test, a vapor trap was positioned around the sample area, flooded with 1× PBS and sealed. The experimental protocol consisted of an amplitude sweep from 0.1% to 100% strain, followed by a frequency sweep at 1 Hz across 15 frequency steps, using an oscillatory strain within the range of 0.01 to 100 s^−1^. The average duration of a full frequency sweep was 45 min ( n=3 per measurement). Measurements at higher frequencies were excluded from the data set due to the structural breakdown of the hydrogels that occurred beyond 10% strain.

The dynamic (or complex) shear modulus ( $G^{*}$ ) is defined in terms of the elastic (or storage, $G^{\prime }$ ) and viscous (or loss, $G^{\prime \prime }$ ) moduli, as follows, where i is the imaginary unit $\left(i=\sqrt{-1}\right)$ :

(1) $$G^{*}\left(\omega \right)=G^{\prime }\left(\omega \right)+iG^{\prime \prime }\left(\omega \right)$$

Subsequently, the magnitude of $G^{*}$ can be obtained by the following equation:

(2) $$G^{*}\left(\omega \right)=\sqrt{{G^{\prime }}^{2}(\omega )+{G^{\prime \prime }}^{2}\;\left(\omega \right)}$$

Therefore, for all the samples, their corresponding plots of modulus versus frequency (*ω*) were obtained from the oscillatory measurements.

### 2.4. Cell Culture Study

Biocompatibility Analysis

Collagen mixtures (2.3 mg/mL) were neutralized at 4 °C to pH 7.4. To induce hydrogel formation, 100 µL of the solution was added to 96-well plates and incubated at 37 °C with 5% CO_2_ for 1.5 h. Once polymerization was complete, a 100 µL suspension of HaCaT cells with >98% cell viability (50,000 cells/well) in low-calcium SFM medium was added to the hydrogel. As a positive control for viability, a HaCaT suspension at the same concentration was cultured as a monolayer. Cell growth was monitored every 48 h ( n=4 ) using the MTS assay (CellTiter 96 AQueous One Solution Reagent, REF. G358A), which measures mitochondrial enzyme activity as an indicator of cellular metabolic activity. Additionally, cell growth was observed by light microscopy.

### 2.5. Skin Substitutes Development

Neutralized collagen AB and CS mixtures (4 °C, 2.3 mg/mL and pH 7.4) were placed into Transwell^®^ supports with a porous polycarbonate membrane (0.3 µm pore size, 25 mm diameter; Celltreat, REF. 230603). The samples were incubated at 37 °C with 5% CO_2_ for 1.5 h to form hydrogels. Subsequently, a suspension of HaCaT cells cultured in low-calcium SFM medium (>98% cell viability) was seeded onto the hydrogels at a concentration of 5 × 10^5^ cells/mL in a total volume of 2 mL. The bottom chamber was filled with fresh low-calcium SFM medium, which was replaced every two days for a total of six days. To induce keratinocyte differentiation, the models were then transferred to an air–liquid interface and cultured for 12 days in Keratinocyte-SFM medium (referred to as SFM stratification medium) (Gibco, REF. 10724-011). This medium was supplemented with 2.4 mM calcium chloride (high calcium), 0.4 μg/mL hydrocortisone, and 5 μg/mL human insulin (all purchased from Sigma-Aldrich, St. Louis, MO, USA).

### 2.6. Histological Analysis

The morphological properties of skin substitutes were evaluated through histological analysis. The samples were fixed at 4 °C using Karnovsky’s buffer for 2 h, then washed with 0.1 M Sodium Cacodylate Buffer (Electron Microscopy Sciences, REF. 12310, Hatfield, PA, USA) for 1 h. Subsequently, samples were incubated in 1% Osmium Tetroxide (EMS, REF. 19100, Hatfield, PA, USA) for 2 h and protected from light. After washing with double-distilled water, samples were dehydrated through graded ethanol concentrations and embedded in Epon resin. Once polymerized, thick sections were prepared and stained with Periodic Acid–Schiff (PAS), then observed under an optical microscope at 20× and 40× objectives. Ultrathin sections were then cut, mounted on copper mesh grids, counterstained with uranyl acetate in methanol and lead citrate, and examined using a JEOL JEM-1010 electron microscope (TEM). The epithelial thickness of the skin substitutes was determined by measuring 120 structures from images of the TEM using the ZEN lite 3.1 Blue Edition software (ZEISS Microscopy, Germany).

### 2.7. Analysis of Cellular Differentiation Markers

RT-PCR was performed to examine the expression levels of differentiation markers in 3D-cultured HaCaT cells. To assess the level of HaCaT differentiation, total RNA was extracted from AB and CS-derived skin substitutes using the GeneJET RNA Purification Kit (Thermo Scientific, REF. 0731, Waltham, MA, USA), according to the manufacturer’s instructions. Genomic DNA was removed using RNAse-free DNase I (Thermo Scientific, REF. EN0521, Waltham, MA, USA), and cDNA was synthesized using the RevertAid H Minus First Strand cDNA Synthesis Kit (Thermo Scientific, REF. K1631, Waltham, MA, USA). The resulting cDNAs were analyzed by endpoint PCR using DreamTaq PCR Master Mix (2X) (Thermo Fisher Scientific, REF. K1071, Waltham, MA, USA). The primers used to amplify cDNAs for keratin 1 (K1), keratin 10 (K10), and involucrin (INV) are listed in Table 1. The PCR amplification followed the protocol described by Micallef et al. (2009) [34].

### 2.8. Statistical Analysis

All analyses were performed with GraphPad Prism v9.0. Data were expressed as mean ± SD. Two-way ANOVA with Tukey’s test was applied to compare and identify statistically significant interactions concerning collagen quality (AB-GLP vs. CS-CMP grade). Comparisons between two groups were analyzed with a Student’s *t*-test. Rheological data were evaluated using linear regression analysis to evaluate the trend of viscoelastic behavior and to compare the parameters adjusted to 10% strain. Statistical significance was set at p<0.05 . In the figures, *p*-values ( p<0.05 ) are indicated as p<0.01(**),p<0.001(***),p<0.0001(**** ).

## 3. Results

### 3.1. Characterization of Collagen Hydrogels

#### 3.1.1. Collagen Hydrogel Fibrillogenesis Kinetics

**Figure 1:**
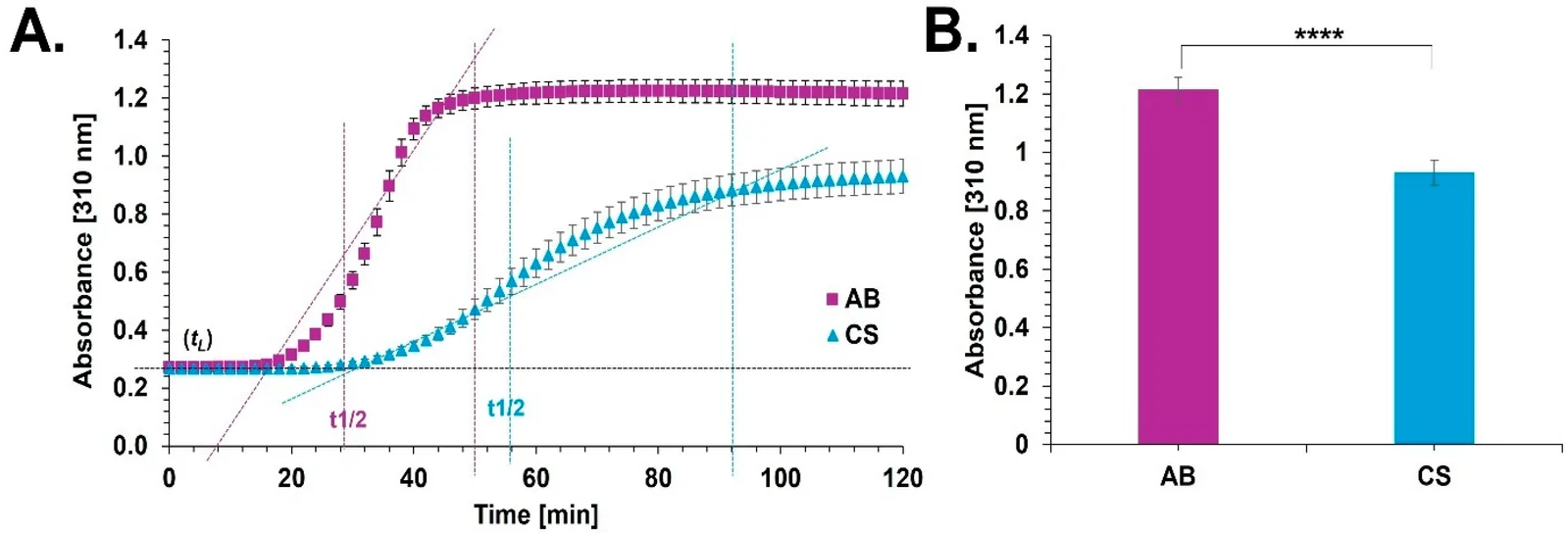
Collagen fibrillogenesis. (**A**) Kinetics. AB: collagen from Advanced Biomatrix. CS: collagen from Collagen Solutions. Kinetics were quantified from spectrophotometric measurements of light absorbance (turbidity), showing distinct lag, exponential growth, and plateau phases. Key parameters such as lag time (*t_L_*) and polymerization half-time (t_1/2_) were extracted from these curves. (**B**) Maximum absorbance obtained at 120 min at 37 °C for AB and CS collagen hydrogels. Data are presented as mean ± SD from 9 measurements. Statistical comparison of mean values between groups was performed using Student’s *t*-Test method, revealing a significant difference ( p=0.0001 [****], DF = 8).

Turbidimetric analysis revealed that both collagen preparations—Advanced Biomatrix (AB, GLP-grade) and Collagen Solutions (CS, GMP-grade)—exhibited characteristic sigmoidal fibrillogenesis curves, consisting of distinct lag, exponential growth, and plateau phases (Figure 1A). Statistically significant differences were observed in maximum absorbance values ( p<0.05 ), with AB exhibiting a higher peak absorbance (1.12 ± 0.04) compared to CS (0.93 ± 0.06) (Figure 1B). The fibrillogenesis rates during the exponential phase ( $\frac{dAbs}{dt}$ ) ranged from 22.3 ± 6.35 × 10^3^ min^−1^ for AB to 47.3 ± 6.35 × 10^3^ min^−1^ for CS (Table 2), indicating a more rapid self-assembly process in the AB collagen. Furthermore, both the average fibrillogenesis time and lag phase duration were shorter for AB hydrogels, reinforcing the observation of faster polymerization kinetics. The final maximum absorbance values differed significantly ( p=0.0001 ), with AB again displaying higher optical density. These findings indicate that fibrillogenesis kinetics and fiber formation efficiency are highly dependent on the collagen quality, despite the identical formulation concentrations (2.3 mg/mL) [23].

#### 3.1.2. Morphological Analysis of Collagen Hydrogels

**Figure 2:**
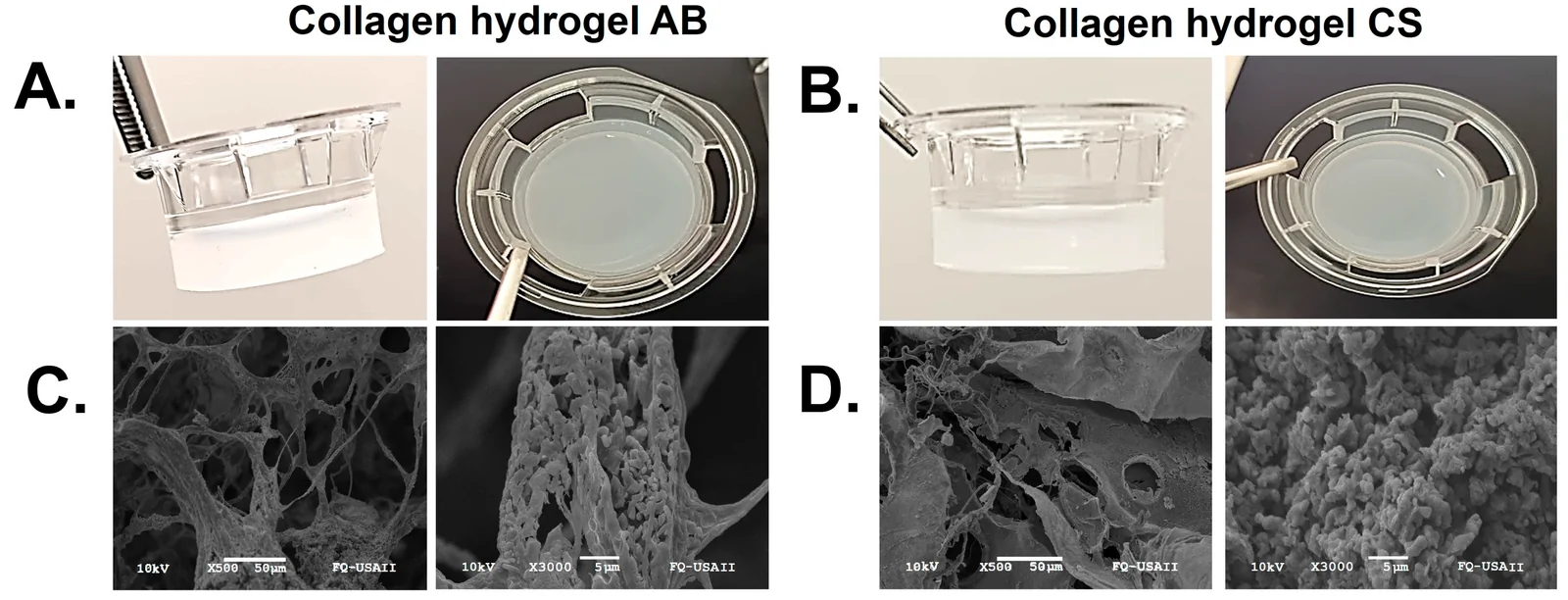
Structural analysis of collagen hydrogels. AB: collagen from Advanced Biomatrix CS: collagen from Collagen Solutions. (**A**,**B**) Crosslinked type I and III bovine collagen hydrogels. (**C**,**D**) Structure of collagen hydrogel by scanning electron microscopy (SEM).

**Figure 3:**
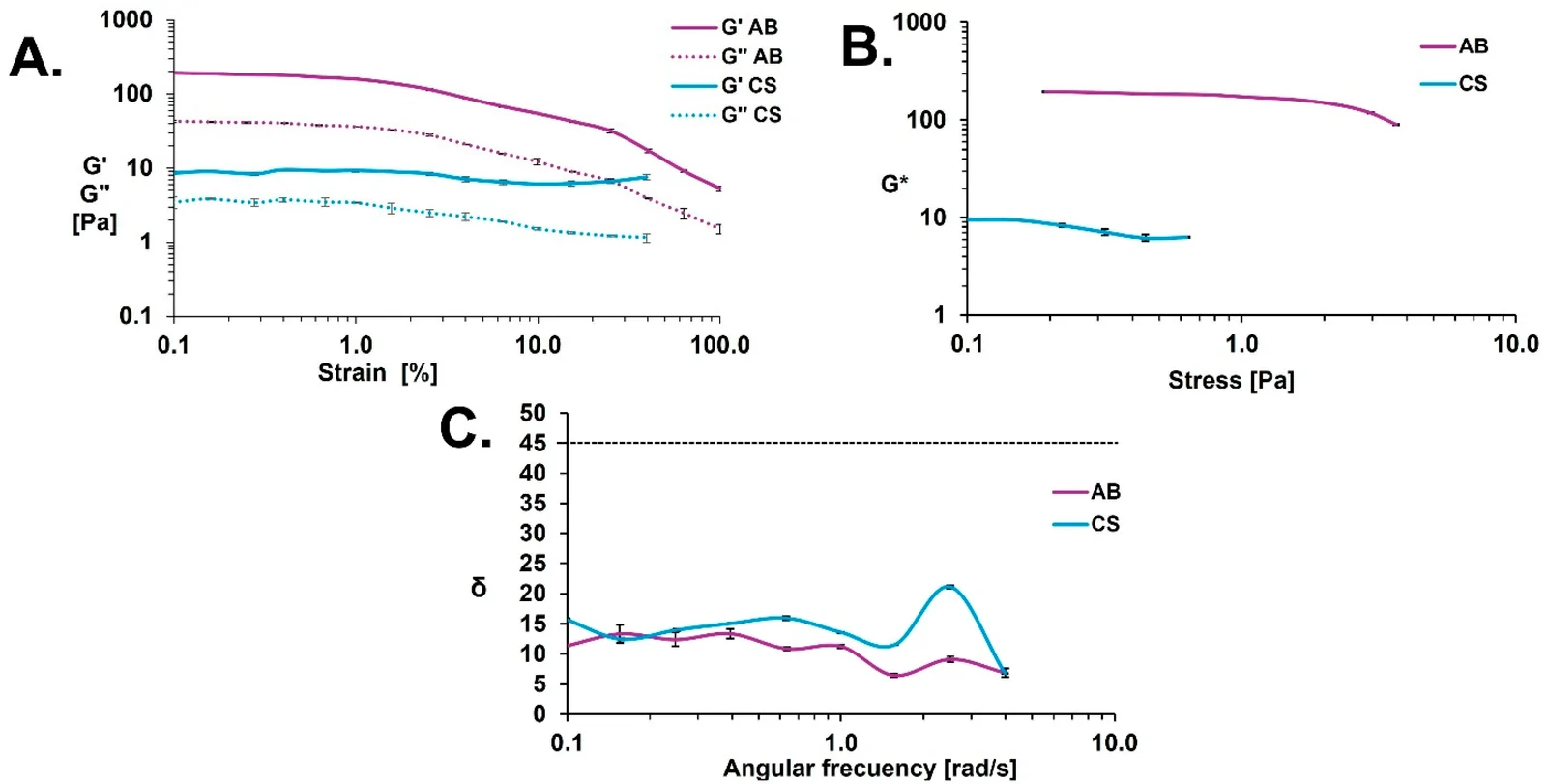
Rheological analysis of collagen hydrogels. Crosslinking of Hydrogels at 37 °C. (**A**) Amplitude scan. (**B**) Complex modules generated from the frequency data. (**C**) Angle obtained from the amplitude data. The data obtained are presented as the mean ± SD from three measurements.

In the 25 mm Transwell^®^ inserts, the collagen hydrogels conformed to the geometry, forming soft, semi-solid, whitish three-dimensional structures. Visually, hydrogels derived from AB collagen exhibited greater turbidity compared with those from CS collagen (Figure 2A,B), suggesting differences in fibrillar density and light-scattering properties. Scanning electron microscopy (SEM) was employed to examine the microstructure of freeze-dried hydrogels at 500× and 3000× magnifications. Subtle yet distinct differences in fibrillar organization were observed, reflecting intrinsic quality differences between the two collagen sources (AB: GLP-grade; CS: GMP-grade). AB-derived hydrogels displayed a highly interconnected fibrillar network, with bundled fibers forming a porous three-dimensional matrix (Figure 2C). This architecture featured heterogeneous pore sizes ranging from 29 ± 11 µm to 80 ± 23 µm, contributing to a structurally denser and mechanically stiffer scaffold (Figure 3B). In contrast, CS-derived hydrogels exhibited a less cohesive microstructure (Figure 2D), characterized by loosely organized fibrils and a more fibrous, fragmented architecture. The pore sizes of CS-derived hydrogels were significantly smaller and more uniform, ranging from 12 ± 3 µm to 26 ± 3 µm, resulting in a weaker and softer matrix (Figure 3B). These findings suggest that collagen quality influences not only fibrillogenesis kinetics, but also the resulting scaffold morphology.

#### 3.1.3. Rheology of Collagen Hydrogels

Hydrogels are inherently viscoelastic materials characterized by a storage modulus (G′) greater than the loss modulus (G″), indicating a predominance of elastic behavior over viscous behavior [18]. In the amplitude graph (Figure 3A), this elastic predominance (G′ > G″) is observed in both types of collagens. However, AB shows significantly higher values of G′ and G″ across the entire deformation range analyzed. This indicates that AB is a considerably stiffer hydrogel, i.e., it has a greater capacity to store elastic energy and is also more viscous with greater energy dissipation than CS, which has much lower G′ and G″ moduli in comparison, indicating a less rigid and viscous structure.

In both hydrogels, the G′ modulus remains relatively constant within the linear region at low strains, a characteristic of well-formed hydrogel networks. The AB hydrogels showed a linear viscoelastic region (LVR) extending up to a strain of 2.54% for G′ and 4.06% for G″ (R^2^ = 0.99), due to their stiffness. In contrast, CS hydrogels exhibited greater deformability, maintaining linearity in G′ up to a strain of 9.96% and in G″ up to a strain of 6.4% (R^2^ = 0.91), as they form a less rigid and viscous structure, allowing for greater energy dissipation under mechanical stress. As deformation increases (>10%), both G′ and G″ decrease, although the decrease is more pronounced in AB than in CS. This suggests a breakdown of the structural network (yielding), and mechanical integrity is lost. For AB, there is a tendency for G′ and G″ to converge more rapidly at high deformations, which could indicate that its network, although more robust, could also collapse rapidly under sufficient stress. In contrast, for CS, G′ and G″ converge but do not cross within the range shown, which would typically indicate that solid behavior is maintained in this range.

Analysis of the complex modulus (G*) confirmed that AB-derived hydrogels are mechanically stronger and stiffer, by approximately one order of magnitude, than CS (almost 10 times higher), maintaining their complex modulus stably under different stress levels until it approaches high values (around 5 Pa), where a drop in G* is observed, indicating that it has much greater overall mechanical resistance, both elastic and viscous (Figure 3B). While CS has a low and relatively constant G* value, indicating a weaker but stable structure under the applied stress range without obvious breakage or creep within the measured range, it maintains a constant stability within the evaluated stress range, albeit it is weaker. Comparatively, CS is more flexible and less rigid, making it suitable for applications where greater molecular mobility, biomolecule diffusion, or a softer matrix is preferred.

Analysis of the complex modulus (Figure 3B) confirmed that AB-derived hydrogels are mechanically stronger and stiffer, whereas CS hydrogels are softer, weaker, and more deformable. A smaller loss modulus G″ corresponded to a lower phase angle (δ). Both matrices exhibited δ between 10° and 20° within the LVR (Figure 3C), well below 45°, indicating solid-like behavior with low energy dissipation, consistent with their predominantly elastic nature.

### 3.2. Cell–Matrix Behavior of Collagen Studies

Biocompatibility Analysis

To evaluate the hydrogels’ biocompatibility, human keratinocytes (HaCaT cell line) were cultured on matrices formed from AB and CS collagen. HaCaT cells were chosen due to their established relevance in skin regeneration and tissue engineering studies [10,35,36,37].

**Figure 4:**
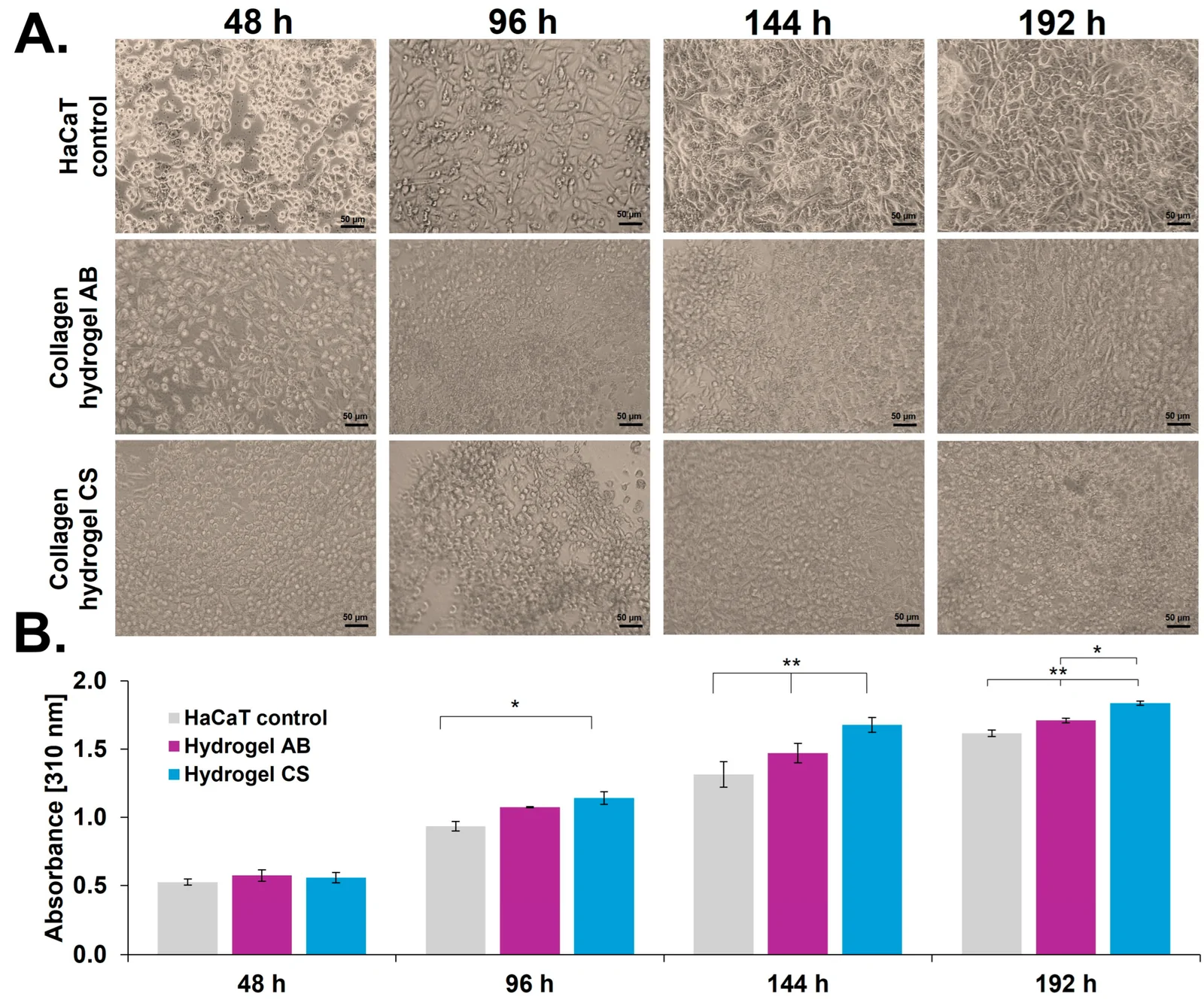
Biocompatibility of Human HaCaT Keratinocytes on Collagen Hydrogels. (**A**) Photographs taken with 10× objectives. Zeiss Axioskop confocal microscope. (**B**) HaCaT cell proliferation on collagen hydrogels. It was determined by measuring cellular mitochondrial enzyme activity (i.e., cellular metabolic activity) by MTS in a 96-well plate. The data obtained and analyzed are presented as the mean ± SD from four measurements. Comparison of mean values between groups and with the control group obtained by ANOVA and Tukey’s method showing a statistically significant difference with *p*-values of 0.01 (*) and 0.001 (**) (DF = 12).

Cell proliferation and viability were monitored over a 192 h period using human keratinocytes (HaCaT cell line) to evaluate hydrogel biocompatibility. Notable differences in cell–matrix interactions were observed between the two hydrogels. Keratinocytes cultured on AB collagen showed elongated morphologies over time (48–192 h), with a significantly larger average diameter of 25.55 µm (Figure 4A), and cell–cell junctions appeared less compact until 144 h. In contrast, cells grown on CS collagen exhibited predominantly circular to cuboidal morphology, with an average cell diameter of 14.53 µm. Additionally, cell–cell junctions appeared more compact and defined in the CS group, suggesting enhanced intercellular adhesion.

Viability assays revealed significantly greater cell proliferation on AB-derived hydrogels compared to CS-derived hydrogels (*p*-values: 0.01 and 0.001, Figure 4B). Cells cultured in AB-derived hydrogels showed increased mitochondrial activity compared to monolayer cultures (HaCaT control), indicating that this collagen source promotes cell proliferation.

These results indicate that the morphological and proliferation responses of keratinocytes are highly dependent on the collagen source. The AB hydrogel promotes higher cell proliferation and a more spread-out morphology, while the CS hydrogel supports a more compact, interconnected cellular network, making it a strong candidate for epidermal tissue engineering [38].

### 3.3. Development of Skin Substitutes and Histological Analysis

**Figure 5:**
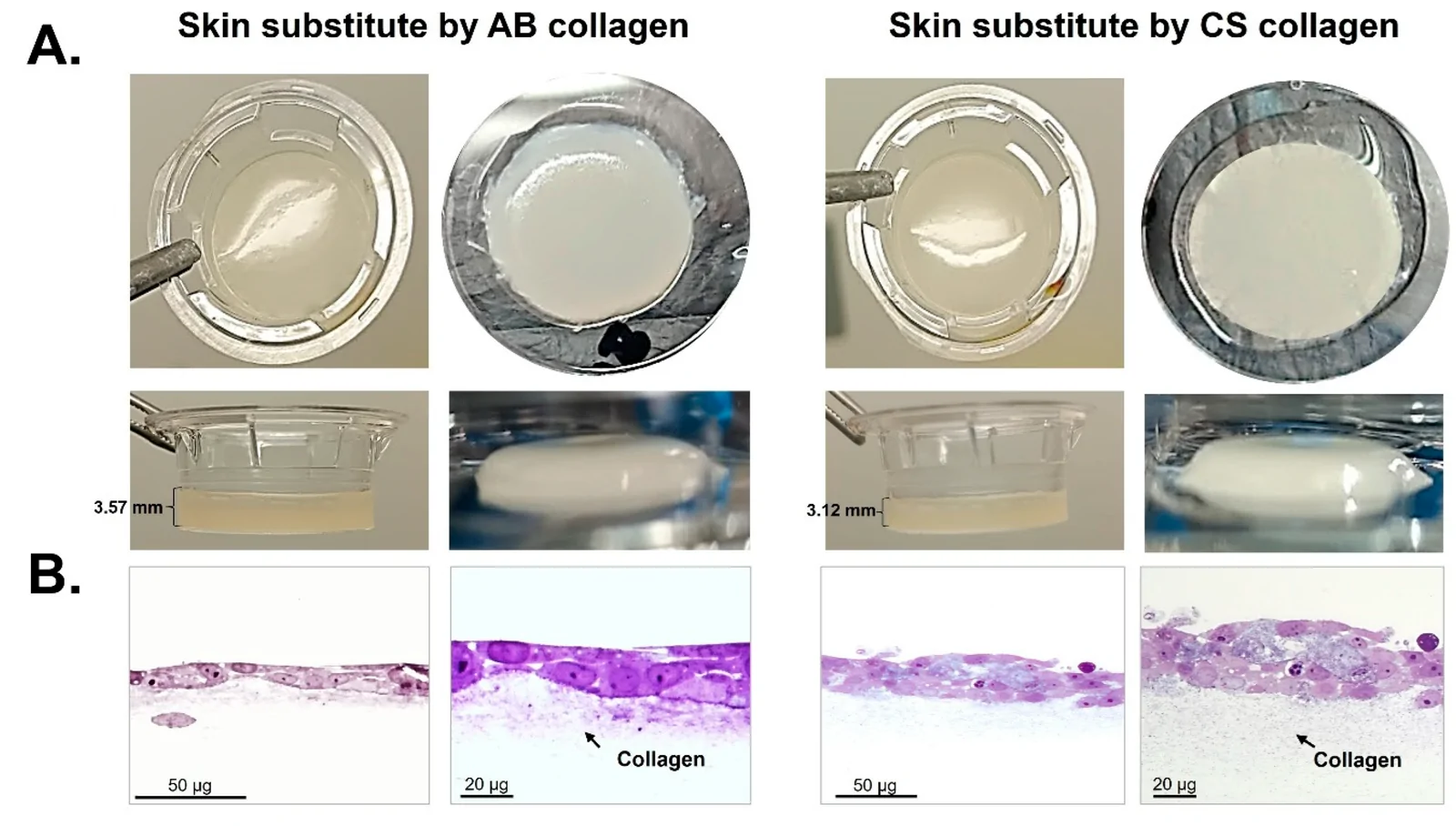
Morphology of skin substitutes developed with AB and CS collagen hydrogels. (**A**) Macroscopic view of full-thickness skin substitutes, showing differences in size, color, and matrix contraction depending on the collagen source (AB vs. CS). (**B**) Cross-sectional histological sections of skin substitutes, stained with Periodic Acid–Schiff (PAS) stain. Images were observed under an optical microscope using 20× and 40× objectives, revealing differences in epidermal architecture and cellular infiltration based on the collagen source.

The use of skin substitutes plays a critical role in skin regeneration by supporting cellular re-epithelialization and accelerating wound healing [39]. After 17 days of culture, circular constructs (25 mm in diameter) were obtained, which showed macroscopic differences depending on the collagen quality (Figure 5A). Substitutes cultured on AB collagen appeared more consistent, with a whitish color and a final thickness of 3.57 mm. In contrast, substitutes generated with CS collagen were softer and slightly thinner (3.12 mm), exhibiting matrix contraction at the periphery, which indicated collagen shrinkage.

Histological analysis (Figure 5B) revealed different epidermal architectures depending on the collagen source. AB-derived substitutes, having a denser and stiffer matrix, supported the formation of keratinocyte layers without cells infiltrating the collagen matrix. In contrast, CS hydrogels, with greater viscoelastic compliance, promoted a multilayered epidermal structure and infiltration of cells into the collagen matrix. This suggests that the more deformable matrix facilitates greater cell integration and stratification. In both conditions, residual collagen was observed, indicating the stability of the substrate throughout the differentiation process.

**Figure 6:**
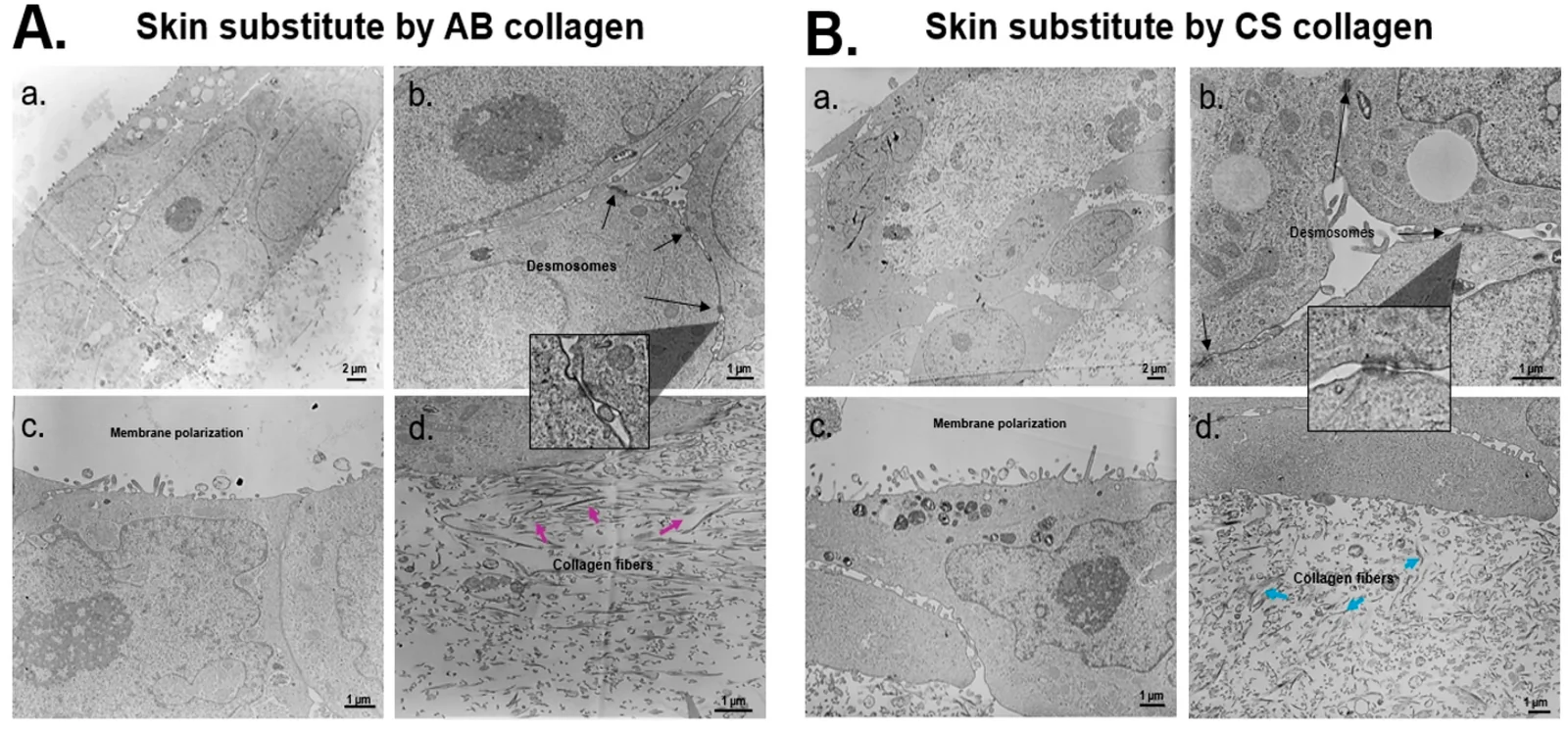
Histological Analysis of Skin Substitutes. Ultrathin sections of skin substitutes were analyzed by TEM. Black arrows show desmosomes connecting cells. Purple and blue arrows show collagen fibers. (**A**) Skin substitute by AB showing tri-layered epithelial sheets, with cells displaying a vertically oriented axis. (**B**) Skin substitute by CS has a thicker, multilayered architecture, with a greater number of epithelial sheets interspersed with extracellular matrix components. (**a**). Different cell layers were generated in skin substitutes and these are shown as evidence of 3D tissue. (**b**). Desmosomal junctions between cells are magnified and shown as evidence of cell differentiation. (**c**). Membrane polarization on the outer layer is shown as microvilli. (**d**). Remaining collagen is shown in skin substitutes.

To obtain more detailed information about keratinocytes’ growth and differentiation on the collagen hydrogels, ultrathin sections of the skin substitutes were examined using a transmission electron microscope (TEM). The images revealed that the constructs generated with AB collagen exhibited trilayered epithelial sheets, with cells displaying a vertically oriented axis (Figure 6A(a)) with an average epithelial thickness of 19.26 ± 4.20 µm (Table S1). Cells adjacent to the collagen matrix (basal level) appeared larger, with defined nuclei. In the outermost layer, cells were smaller and presented more compact nuclei, reduced cell polarization, and a low presence of mitochondria (Figure 6A(c)). Desmosomal junctions between adjacent keratinocytes were also evident (Figure 6A(b)).

In comparison, skin substitutes formed with CS collagen showed a thicker, multilayered architecture, with a greater number of epithelial sheets interspersed with extracellular matrix components (Figure 6B(a)), with a higher average epithelial thickness (42.39 ± 4.27 µm) (Table S2). At the basal level, cells showed an organized structure with vertically aligned axes and prominent nuclei. Toward the outermost layers, keratinocytes appeared more elongated and adopted an irregular polyhedral morphology. Desmosomal junctions were evident in this region (Figure 6B(b)), and keratinocytes showed more pronounced cellular polarization and a higher mitochondrial density. These features are indicative of active cytoplasmic processes reminiscent of those in the stratum spinosum (Figure 6B(c)). In both cases, the observed morphological differences, consistent with mature intercellular adhesion structures, confirmed that stratified epithelia are generated [40,41]. The results further suggest a more advanced state of cellular differentiation in the CS-based substitutes.

The residual collagen matrix revealed marked differences in fibrillar organization between the two conditions. The AB hydrogels retained a denser and more abundant fibrillar network, whereas the CS matrix exhibited a visibly reduced number of collagen fibers (Figure 6A(d),B(d)). This observation suggests a greater degree of collagen degradation in the CS substitutes, potentially driven by enhanced cell–matrix interactions and enzymatic remodeling associated with higher keratinocyte activity.

Cellular Differentiation Markers of HaCaT in Skin Substitutes

**Figure 7:**
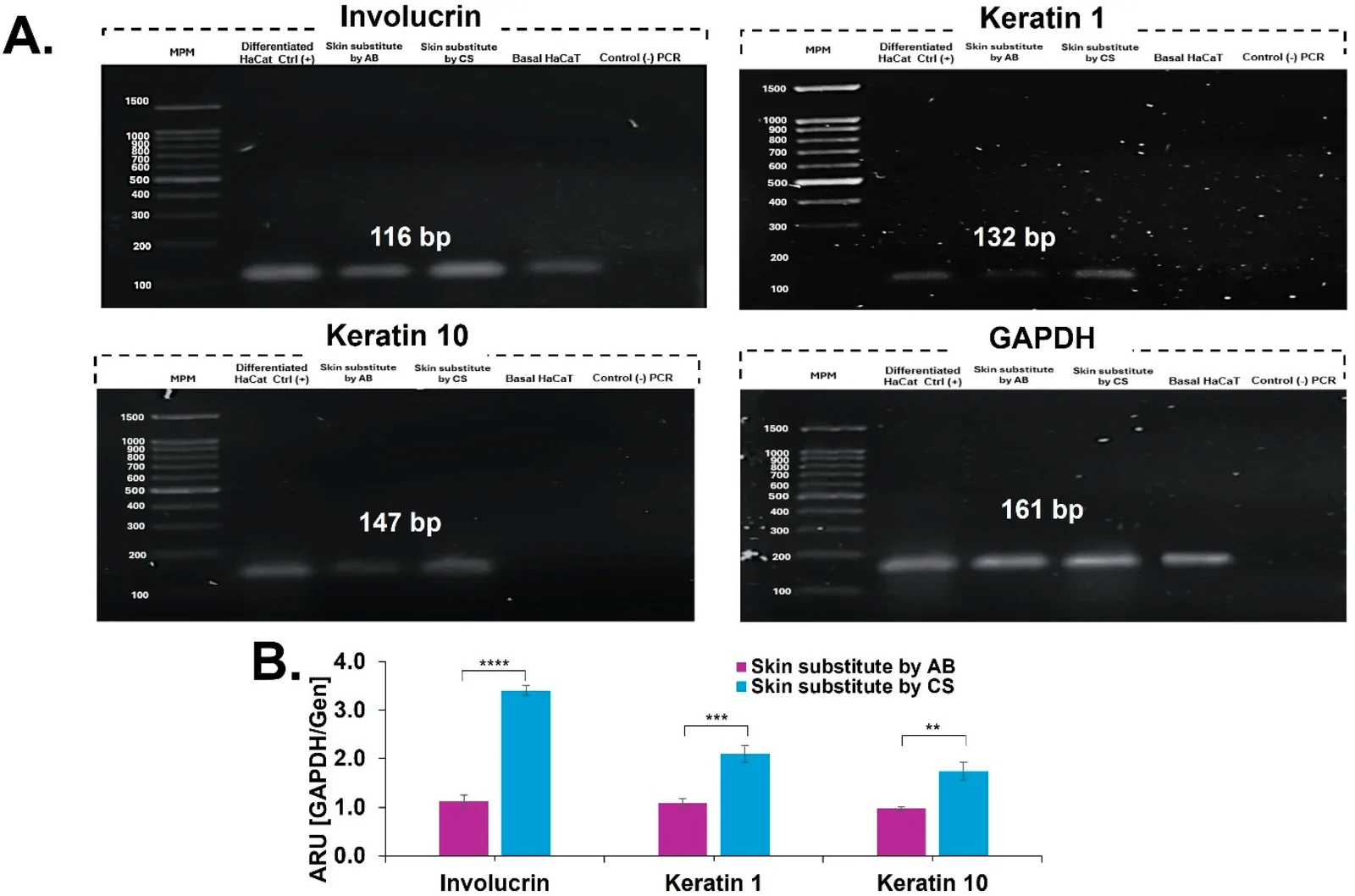
Skin substitutes differentiation analysis for differentiation markers K1, K10 and involucrin. (**A**) Representative gel electrophoresis results from endpoint RT-PCR. (**B**) Densitometric analysis of differentiated skin substitutes. For each marker, mRNA levels were normalized with a housekeeping gene (GAPDH). Data obtained are shown as mean ± SD from three measurements. Statistical comparison of mean values between groups was obtained using the *t*-Student method showing a statistically significant difference with *p*-values of 0.01 (**), 0.001 (***) and 0.0001 (****) (DF: 3, *p* < 0.05).

To assess the degree of keratinocyte differentiation, mRNA expression levels of keratin 1 (K1), keratin 10 (K10), and involucrin—established markers of epidermal maturation [42]—were quantified in skin substitutes generated with both collagen types. Reverse transcription PCR (RT-PCR) analysis revealed statistically significant (*p*-values of 0.01, 0.001, and 0.0001) increases in the expression of these markers in skin substitutes derived from CS hydrogels compared to those derived from AB hydrogels (Figure 7B). This finding of enhanced keratinization is visually supported by the raw RT-PCR gel electrophoresis data (Figure 7A), where the intensity of the bands for K1, K10, and involucrin is visibly higher in the CS condition. GAPDH was used as a constitutive internal control, and HaCaT cells cultured in SFM stratification medium served as a positive control for differentiation. These results suggest that the mechanical properties of the CS hydrogel—characterized by greater compliance and deformation tolerance—foster a microenvironment that more effectively supports keratinocyte differentiation. This highlights the critical role of scaffold viscoelasticity in facilitating epidermal stratification and the maturation of functional tissue.

## 4. Discussion

Collagen hydrogels possess a remarkable capacity to mimic the structure of the extracellular matrix (ECM), establishing them as pivotal scaffolding materials in tissue engineering and regenerative medicine [1,43]. Although certain studies suggest that unmodified collagen hydrogels may lack the complexity required for supporting dermal equivalent formation [44,45,46], our findings demonstrate that Type I collagen hydrogels not only sustain robust keratinocyte proliferation but also actively promote their differentiation, resulting in the generation of functional skin substitutes. Achieving this outcome depends heavily on the physical and mechanical properties of the hydrogel, as 3D scaffolds play an important role in mediating the cell–matrix interactions essential for tissue development [17]. Key properties, such as stiffness, pore size, viscoelasticity, and fibrillar architecture, are highly tunable and are influenced by multiple manufacturing parameters, such as collagen source, purification method, polymerization conditions, and pH [23].

In this study, two commercially available bovine Type I collagens with distinct production standards (GMP-grade Collagen Solutions and GLP-grade Advanced Biomatrix) were selected to evaluate how manufacturing variables impact hydrogel mechanics and, in turn, modulate keratinocyte adhesion, stratification, and differentiation. The results demonstrate that collagen quality significantly influences hydrogel architecture and viscoelastic properties, thereby shaping cellular behavior through mechanotransduction pathways [47]. These findings underscore the importance of rational design and standardization in collagen scaffold development to optimize regenerative outcomes.

Type I collagen undergoes a self-assembly process known as fibrillogenesis, wherein individual triple-helical molecules align and crosslink to form fibrillar structures that give rise to viscoelastic hydrogels with distinct mechanical and morphological properties [48,49]. Turbidimetric and structural analyses (Figure 1 and Figure 2) revealed significant differences in fibril formation between the two collagen types, particularly in terms of maximum turbidity and fibrillar architecture. These variations could be from post-translational modifications within the collagen chains. Such modifications enhance inter- and intra-molecular crosslinking, thereby influencing fibril stability, organization, and mechanical behavior [15]. Importantly, both collagen types are specified as a mixture of atelocollagen (lacking telopeptides) and telocollagen (retaining telopeptides) in different proportions [50], which likely contributes to the observed fibrillar differences. This is particularly relevant since hydroxylysine residues, predominantly located in the telopeptide regions rather than in the central triple-helical domain, are essential for enzymatic crosslinking and stabilization of the fibrillar network [17]. The presence or absence of telopeptides may therefore significantly influence the kinetics of fibrillogenesis and the mechanical robustness of the resulting hydrogel scaffold [51].

Cell–substrate interactions are strongly influenced by the mechanical properties of the scaffold, particularly its stiffness. Among these properties, the compressive modulus plays a critical role in the design of engineered scaffolds, as it modulates key cellular behaviors such as adhesion, morphology, migration, and differentiation [52]. This study established a clear correlation between the fibrillar architecture of the collagen hydrogels, their deformation tolerance, and the HaCaT cells’ morphology during skin substitute generation. AB-derived hydrogels exhibited a more organized fibrillar network, along with higher storage (G′) and loss (G″) moduli (Figure 3), and a greater complex modulus, indicative of a considerably stiffer hydrogel, meaning that it has a greater capacity to store elastic energy and is also more viscous. In contrast, CS-driven hydrogels showed lower G′ and G″ values but are more flexible, less rigid, and suitable for applications where greater molecular mobility, diffusion of biomolecules, or a softer matrix is preferred. Pioneering work by Engler et al. (2006) demonstrated that when hydrogel stiffness closely mimics the mechanical properties of a cell’s native microenvironment, it can direct lineage-specific differentiation and shape cell morphology and fate [53]. These findings underscore the pivotal role of the extracellular matrix (ECM) in guiding tissue development [54].

Chaudhuri (2020) and Cao (2018) have highlighted how the physicochemical properties of the extracellular matrix continuously influence cellular processes [47,55]. The disorganized fibrillar structure of collagen and the presence of surface polar functional groups such as carbonyl and carboxyl groups appear to be key factors in promoting cell–matrix interactions [47], particularly within atelocollagen-based matrices. According to Sarrigiannidis et al. (2021), collagen fibrils are enriched with adhesion motifs such as collagen triple-helix ligands (GxOGER) and the arginine–glycine–aspartic acid (RGD) sequence, which interact with integrin receptors like α1β1 and α2β1 on keratinocyte membranes [17]. Furthermore, non-covalent interactions, including hydrogen bonding and van der Waals forces, are known to enhance cell–matrix adhesion, thereby improving biocompatibility and promoting cellular anchorage and proliferation on collagen hydrogels [38]. The proliferation of HaCaT cells and formation of homogeneous monolayers on both types of hydrogels (Figure 4) confirm the biocompatibility and structural suitability of these collagen matrices for epidermal reconstruction.

A uniform basal monolayer is crucial for epidermal stratification; irregular keratinocyte colonies can lead to uneven stratification, potential detachment, or incomplete layering [56]. During skin substitute development, HaCaT cells successfully adhered to both AB and CS collagen hydrogels and formed a continuous monolayer after approximately 7 days in submerged culture, as evidenced by the formation of a thin cell sheet at 192 h (Figure 4). Following basal layer formation, stratification was induced by elevating extracellular calcium concentrations and exposing the cultures to an air–liquid interface—conditions known to trigger keratinocyte differentiation [57]. This process led to the formation of whitish, circular skin substitutes composed of stratified and differentiated keratinocyte layers. AB-based constructs were more resistant to handling (Figure 5A) but supported the formation of fewer cell sheets (Figure 6A). In contrast, CS-based substitutes (Figure 5B), though mechanically less resistant, promoted the development of multilayer cell sheets (Figure 6B) and exhibited a statistically significantly higher degree of cell differentiation (Figure 7). This difference in cell layer development and level of differentiation may stem from cell–matrix interactions mediated by integrins, which transduce and integrate mechanical signals [14,17].

The amount of tension generated by cell–matrix junctions is directly related to hydrogels’ ability to tolerate deformation, as described by Martino et al. (2018) [49]. It appears that CS hydrogels, with their higher deformability, support greater cell proliferation and differentiation. According to Zigon-Branc et al. (2019), the composition of the extracellular matrix regulates the precise expression of integrin subsets, which, when coupled with different signaling cascades, induce specific cellular responses such as cell proliferation and differentiation [58].

## 5. Conclusions

This study demonstrates that bovine collagen hydrogels are promising 3D scaffolds for the development of skin substitutes. A key distinction lies in their collagen composition and crosslinking: AB hydrogels contain both atelocollagen and telocollagen, facilitating enhanced fiber rearrangement through natural crosslinking. In contrast, CS hydrogels, composed solely of atelocollagen, exhibit limited crosslinking and form a more heterogeneous fibrillar network.

These structural differences influence the viscoelastic behavior of the hydrogels, which, in turn, affect cell–substrate interactions. Notably, the more deformable and compliant CS hydrogels better mimic the native extracellular matrix, providing a porous environment that supports enhanced keratinocyte stratification and the formation of a compact cellular network. Although mechanically weaker, CS hydrogels support the formation of multilayered epithelial structures with a higher degree of cellular differentiation, highlighting their potential in skin tissue engineering applications.

## Figures and Tables

**Table 1:** Expression of terminal differentiation markers at the mRNA Level in skin substitutes.

Marker	Primer Pair	Genbank Accession No.
K 1	FP: 5′-ATTTCTGAGCTGAATCGTGTGATC-3′ RP: 5′-CTTGGCATCCTTGAGGGCATT-3′	BC063697
K 10	FP: 5′-TGATGTGAATGTGGAAATGAATGC-3′ RP: 5′-GTAGTCAGTTCCTTGCTCTTTTCA-3′	NM_000421
Involucrin	FP: 5′-GGGTGGTTATTTATGTTTGGGTGG-3′ RP: 5′-GCCAGGTCCAAGACATTCAAC-3′	BC046391

**Table 2:** Collagen polymerization kinetic parameters.

Commercial Brand	Advanced Biomatrix	Collagen Solutions
Lag time *t_L_* (min)	22	34
Polymerization half-time t_1/2_ (min)	34 ± 6.1	58 ± 2.1
Polymerization rate during growth phase (10^−3^/min)	22.3 ± 6.4	47.3 ± 6.3

## Data Availability

The raw data supporting the conclusions of this article will be made available by the authors on request.

## Supplementary Materials

The following supporting information can be downloaded at: https://www.mdpi.com/article/10.3390/polym17172325/s1, Table S1: Epithelial Thickness by Advanced Biomatrix. Table S2: Epithelial Thickness by Collagen Solutions.

## References

[1] Mantha S., Pillai S., Khayambashi P., Upadhyay A., Zhang Y. (2019). Smart Hydrogels in Tissue Engineering and Regenerative Medicine. Materials.

[2] Sawadkar P., Lali F., Garcia-Gareta E., Gil Garrido B., Chaudhry A., Matharu P., Kyriakidis C., Greco K. (2025). Innovative hydrogels in cutaneous wound healing: Current status and future perspectives. Front. Bioeng. Biotechnol..

[3] Perez-Favila A., Martinez-Fierro M.L., Rodriguez-Lazalde J.G., Cid-Baez M.A., Zamudio-Osuna M.d.J., Martinez-Blanco M.d.R., Mollinedo-Montaño F.E., Rodriguez-Sanchez I.P., Castañeda-Miranda R., Garza-Veloz I. (2019). Current therapeutic strategies in diabetic foot ulcers. Medicina.

[4] Kondej K., Zawrzykraj M., Czerwiec K., Deptuła M., Tymińska A., Pikuła M. (2024). Bioengineering Skin Substitutes for Wound Management—Perspectives and Challenges. Int. J. Mol. Sci..

[5] Maarof M., Nadzir M.M., Mun L.S., Fauzi M.B., Chowdhury S.R., Idrus R.B.H., Lokanathan Y. (2021). Hybrid Collagen Hydrogel/Chondroitin-4-Sulphate Fortified with Dermal Fibroblast Conditioned Medium for Skin. Polymers.

[6] Vecin N.M., Kirsner R.S. (2023). Skin substitutes as treatment for chronic wounds: Current and future directions. Front. Med..

[7] Haldar S., Sharma A., Gupta S., Chauhan S., Roy P., Lahiri D. (2019). Bioengineered smart trilayer skin tissue substitute for efficient deep wound healing. Mater. Sci. Eng. C.

[8] Nicholas M.N., Yeung J. (2017). Current status and future of skin substitutes for chronic wound healing. J. Cutan. Med. Surg..

[9] Ciulla M.G., Massironi A., Sugni M., Ensign M.A., Marzorati S., Forouharshad M. (2023). Recent Advances in the Development of Biomimetic Materials. Gels.

[10] García-Cerna S., Sánchez-Pacheco U., Meneses-Acosta A., Rojas-García J., Campillo-Illanes B., Segura-González D., Peña-Malacara C. (2022). Evaluation of Poly-3-Hydroxybutyrate (P3HB) Scaffolds Used for Epidermal Cells Growth as Potential Biomatrix. Polymers.

[11] Golinski P., Menke H., Hofmann M., Valesky E., Butting M., Kippenberger S., Bereiter-Hahn J., Bernd A., Kaufmann R., Zoeller N.N. (2015). Development and characterization of an engraftable tissue-cultured skin autograft: Alternative treatment for severe electrical injuries?. Cells Tissues Organs.

[12] Walimbe T., Calve S., Panitch A., Sivasankar M.P. (2019). Incorporation of types I and III collagen in tunable hyaluronan hydrogels for vocal fold tissue engineering. Acta Biomater..

[13] Montalbano G., Toumpaniari S., Popov A., Duan P., Chen J., Dalgarno K., Scott W., Ferreira A. (2018). Synthesis of bioinspired collagen/alginate/fibrin based hydrogels for soft tissue engineering. Mater. Sci. Eng. C.

[14] Tracy L.E., Minasian R.A., Caterson E.J. (2016). Extracellular Matrix and Dermal Fibroblast Function in the Healing Wound. Adv. Wound Care.

[15] Maher M.K., White J.F., Glattauer V., Yue Z., Hughes T.C., Ramshaw J.A.M., Wallace G.G. (2022). Variation in Hydrogel Formation and Network Structure for Telo-, Atelo-and Methacrylated Collagens. Polymers.

[16] Dong C., Lv Y. (2016). Application of collagen scaffold in tissue engineering: Recent advances and new perspectives. Polymers.

[17] Sarrigiannidis S.O., Rey J.M., Dobre O., González-García C., Dalby M.J., Salmeron-Sanchez M. (2021). A tough act to follow: Collagen hydrogel modifications to improve mechanical and growth factor loading capabilities. Mater. Today Bio.

[18] Stojkov G., Niyazov Z., Picchioni F., Bose R.K. (2021). Relationship between structure and rheology of hydrogels for various applications. Gels.

[19] Hameed P., Manivasagam G. (2021). An overview of bio-actuation in collagen hydrogels: A mechanobiological phenomenon. Biophys. Rev..

[20] Walimbe T., Panitch A. (2020). Best of both hydrogel worlds: Harnessing bioactivity and tunability by incorporating glycosaminoglycans in collagen hydrogels. Bioengineering.

[21] Figueiredo M.P., Rodríguez-Fernández S., Copes F., Mantovani D. (2025). Review of collagen type I-based hydrogels: Focus on composition- structure-properties relationships. npj Biomed. Innov..

[22] Osidak E.O., Kalabusheva E.P., Alpeeva E.V., Belousov S.I., Krasheninnikov S.V., Grigoriev T.E., Domogatsky S.P., Vorotelyak E.A., Chermnykh E.S. (2021). Concentrated collagen hydrogels: A new approach for developing artificial tissues. Materialia.

[23] Antoine E.E., Vlachos P.P., Rylander M.N. (2014). Review of collagen i hydrogels for bioengineered tissue microenvironments: Characterization of mechanics, structure, and transport. Tissue Eng. Part B Rev..

[24] Chelu M., Calderon Moreno J.M., Musuc A.M., Popa M. (2024). Natural Regenerative Hydrogels for Wound Healing. Gels.

[25] Bindi B., Perioli A., Melo P., Mattu C., Ferreira A.M. (2023). Bioinspired Collagen/Hyaluronic Acid/Fibrin-Based Hydrogels for Soft Tissue Engineering: Design, Synthesis, and In Vitro Characterization. J. Funct. Biomater..

[26] Sánchez-Cid P., Alonso-González M., Jiménez-Rosado M., Benhnia M.R.-E., Ruiz-Mateos E., Ostos F.J., Romero A., Perez-Puyana V.M. (2024). Effect of different crosslinking agents on hybrid chitosan/collagen hydrogels for potential tissue engineering applications. Int. J. Biol. Macromol..

[27] Ramasamy S., Davoodi P., Vijayavenkataraman S., Teoh J.H., Thamizhchelvan A.M., Robinson K.S., Wu B., Fuh J.Y., DiColandrea T., Zhao H. (2021). Optimized construction of a full thickness human skin equivalent using 3D bioprinting and a PCL/collagen dermal scaffold. Bioprinting.

[28] Gershlak J.R., Hernandez S., Fontana G., Perreault L.R., Hansen K.J., Larson S.A., Binder B.Y., Dolivo D.M., Yang T., Dominko T. (2017). Crossing kingdoms: Using decellularized plants as perfusable tissue engineering scaffolds. Biomaterials.

[29] Antoine E.E., Vlachos P.P., Rylander M.N. (2015). Tunable collagen I hydrogels for engineered physiological tissue micro-environments. PLoS ONE.

[30] Kamaruzaman N., Fauzi M.B., Tabata Y., Yusop S.M. (2023). Functionalised Hybrid Collagen-Elastin for Acellular Cutaneous Substitute Applications. Polymers.

[31] Fan L., Ren Y., Emmert S., Vučković I., Stojanovic S., Najman S., Schnettler R., Barbeck M., Schenke-Layland K., Xiong X. (2023). The Use of Collagen-Based Materials in Bone Tissue Engineering. Int. J. Mol. Sci..

[32] Advanced Biomatrix (2019). Certificate of Analysis PureCol.

[33] Collagen Solutions (2016). Protocol: Collagen Solutions;.

[34] Micallef L., Belaubre F., Pinon A., Jayat-Vignoles C., Delage C., Charveron M., Simon A. (2009). Effects of extracellular calcium on the growth-differentiation switch in immortalized keratinocyte HaCaT cells compared with normal human keratinocytes. Exp. Dermatol..

[35] Lundvig D.M.S., Pennings S.W.C., Brouwer K.M., Mtaya-Mlangwa M., Mugonzibwa E., Kuijpers-Jagtman A.M., Wagener F.A., Hoff J.W.V.D. (2015). Cytoprotective responses in HaCaT keratinocytes exposed to high doses of curcumin. Exp. Cell Res..

[36] Tao K., Bai X.Z., Zhang Z.F., Shi J., Hu X., Tang C., Hu D., Han J. (2013). Construction of the tissue engineering seed cell (HaCaT-EGF) and analysis of its biological characteristics. Asian Pac. J. Trop. Med..

[37] Maas-Szabowski N., Stärker A., Fusenig N.E. (2003). Epidermal tissue regeneration and stromal interaction in HaCaT cells is initiated by TGF-α. J. Cell Sci..

[38] Fujisaki H., Futaki S., Yamada M., Sekiguchi K., Hayashi T., Ikejima T., Hattori S. (2018). Respective optimal calcium concentrations for proliferation on type I collagen fibrils in two keratinocyte line cells, HaCaT and FEPE1L-8. Regen. Ther..

[39] Raina N., Rani R., Pahwa R., Gupta M. (2020). Biopolymers and treatment strategies for wound healing: An insight view. Int. J. Polym. Mater. Polym. Biomater..

[40] Moroki T. (2023). Morphological characteristics and notes of the skin in preclinical toxicity assessment. J. Toxicol. Pathol..

[41] Bonifant H., Holloway S. (2019). A review of the effects of ageing on skin integrity and wound healing. Br. J. Community Nurs..

[42] Jung M.H., Jung S.M., Shin H.S. (2016). Co-stimulation of HaCaT keratinization with mechanical stress and air-exposure using a novel 3D culture device. Sci. Rep..

[43] Zhang M., Xing J., Zhong Y., Zhang T., Liu X., Xing D. (2024). Advanced function, design and application of skin substitutes for skin regeneration. Mater. Today Bio.

[44] Thongchai K., Chuysinuan P., Thanyacharoen T., Techasakul S., Ummartyotin S. (2020). Characterization, release, and antioxidant activity of caffeic acid-loaded collagen and chitosan hydrogel composites. J. Mater. Res. Technol..

[45] Zhang C., Yang X., Hu W., Han X., Fan L., Tao S. (2020). Preparation and characterization of carboxymethyl chitosan/collagen peptide/oxidized konjac composite hydrogel. Int. J. Biol. Macromol..

[46] Ghorbani M., Roshangar L. (2019). Construction of collagen/nanocrystalline cellulose based-hydrogel scaffolds: Synthesis, characterization, and mechanical properties evaluation. Int. J. Polym. Mater. Polym. Biomater..

[47] Cao H., Duan L., Zhang Y., Cao J., Zhang K. (2021). Current hydrogel advances in physicochemical and biological response-driven biomedical application diversity. Signal Transduct. Target. Ther..

[48] Kaczmarek-Szczepańska B., Polkowska I., Małek M., Kluczyński J., Paździor-Czapula K., Wekwejt M., Michno A., Ronowska A., Pałubicka A., Nowicka B. (2023). The characterization of collagen-based scaffolds modified with phenolic acids for tissue engineering application. Sci. Rep..

[49] Martino F., Perestrelo A.R., Vinarský V., Pagliari S., Forte G. (2018). Cellular mechanotransduction: From tension to function. Front. Physiol..

[50] Advanced Biomatrix (2017). PureCol®. 9007-34-5. https://advancedbiomatrix.com/purecol/.

[51] Terajima M., Taga Y., Nakamura T., Guo H.-F., Kayashima Y., Maeda-Smithies N., Parag-Sharma K., Kim J.S., Amelio A.L., Mizuno K. (2022). Lysyl hydroxylase 2 mediated collagen post-translational modifications and functional outcomes. Sci. Rep..

[52] Caliari S.R., Burdick J.A. (2016). A Practical Guide to Hydrogels for Cell Culture. Nat Methods..

[53] Engler A.J., Sen S., Sweeney H.L., Discher D.E. (2006). Matrix Elasticity Directs Stem Cell Lineage Specification. Cell.

[54] Colombo I., Sangiovanni E., Maggio R., Mattozzi C., Zava S., Corbett Y., Fumagalli M., Carlino C., Corsetto P.A., Scaccabarozzi D. (2017). HaCaT Cells as a Reliable in Vitro Differentiation Model to Dissect the Inflammatory/Repair Response of Human Keratinocytes. Mediat. Inflamm..

[55] Chaudhuri O., Cooper-White J., Janmey P.A., Mooney D.J., Shenoy V.B. (2020). Effects of extracellular matrix viscoelasticity on cellular behaviour. Nature.

[56] Zhao X., Lang Q., Yildirimer L., Lin Z.Y., Cui W., Annabi N., Ng K.W., Dokmeci M.R., Ghaemmaghami A.M., Khademhosseini A. (2016). Photocrosslinkable Gelatin Hydrogel for Epidermal Tissue Engineering. Adv. Healthc. Mater..

[57] Cho J., Bejaoui M., Isoda H. (2025). Regulation of keratinocyte proliferation and differentiation by secoiridoid oleacein in monoculture and fibroblast co-culture models. Biomed Pharmacother. Biomed. Pharmacother..

[58] Žigon-Branc S., Markovic M., Van Hoorick J., Van Vlierberghe S., Dubruel P., Zerobin E., Baudis S., Ovsianikov A. (2019). Impact of Hydrogel Stiffness on Differentiation of Human. Tissue Eng. Part A.

