# Supplementary material for: The Role of Collagen Rheology in Human Keratinocyte Differentiation: Implications for Skin Substitute Development
Source: Polymers (Basel). 2025 Aug 28;17(17):2325. doi: 10.3390/polym17172325 (PMC12430946; doi:10.3390/polym17172325)
Supplement: Supplementary file 1 [file polymers-17-02325-s001.zip › polymers-3798729-supplementary.pdf]

Table S1. Epithelial Thickness by Advanced Biomatrix.

Measurements	Micrography 1 (µM)	Micrography 2 (µM)	Micrography 3 (µM)	Micrography 4 (µM)
1	17.54	21.17	26.68	18.83
2	14.78	21.66	25.64	19.49
3	14.78	24.53	14.69	25.60
4	15.05	22.56	18.80	20.38
5	18.83	19.64	19.02	21.56
6	14.32	22.64	19.49	22.51
7	15.67	22.06	13.41	18.83
8	13.67	22.56	17.84	19.49
9	15.42	15.70	24.29	25.60
10	19.49	17.84	22.56	25.97
11	17.60	24.29	19.64	22.66
12	21.69	26.63	23.16	18.82
13	20.70	26.68	19.99	21.51
14	25.60	25.64	19.33	21.33
15	15.56	23.16	20.62	15.05
16	11.50	19.99	13.06	14.32
17	25.97	14.77	13.20	0.00
18	14.53	14.69	17.68	22.50
19	22.66	18.80	18.90	21.78
20	21.38	19.02	14.78	25.60
21	17.84	19.49	15.05	11.50
22	20.62	13.41	14.32	25.97
23	26.63	16.09	13.67	14.53
24	26.68	16.80	17.60	22.58
25	25.64	13.06	20.70	25.97
26	23.16	13.20	16.09	22.66
27	17.84	14.85	22.26	18.82
28	20.62	17.68	20.87	14.53
29	26.63	18.90	13.67	22.66
30	20.62	19.33	20.92	21.38
**Average**	19.43	19.56	18.60	19.23
**Standard Deviation (SD)**	4.5	4.0	3.8	4.5
**Global average**	**19.26 µM**			
**Global SD**	**4.20 µM**			

Table S2. Epithelial Thickness by Collagen Solutions.

Measurements	Micrography 1 (μM)	Micrography 2 (μM)	Micrography 3 (μM)	Micrography 4 (μM)
1	39.60	47.51	34.75	39.49
2	36.31	47.71	35.36	40.21
3	41.03	47.72	37.26	40.24
4	38.66	47.53	46.54	41.05
5	36.77	46.96	47.12	40.42
6	39.13	48.72	47.51	39.92
7	41.96	48.73	47.71	39.12
8	40.20	47.43	36.70	39.16
9	37.84	48.65	32.45	38.85
10	37.85	48.36	33.41	39.23
11	36.70	48.79	33.79	38.58
12	32.45	50.34	50.68	39.07
13	33.41	47.83	51.09	40.57
14	33.79	50.68	52.14	40.92
15	34.75	51.09	38.66	41.17
16	35.36	52.14	36.77	41.02
17	37.26	50.94	39.13	40.82
18	38.04	50.25	39.60	40.76
19	38.03	49.30	36.31	40.44
20	39.00	46.74	41.03	40.90
21	42.50	47.33	47.43	40.10
22	43.84	46.15	48.65	40.93
23	46.15	45.96	48.36	40.35
24	46.92	46.36	48.79	37.73
25	47.12	41.90	50.34	38.04
26	46.92	40.93	47.83	38.03
27	46.72	40.35	39.12	39.00
28	45.56	37.73	39.16	42.50
29	46.54	38.54	38.85	46.92
30	47.12	37.71	39.23	47.71
**Average**	40.25	46.68	42.19	40.44
**Standard Deviation (SD)**	4.7	4.0	6.2	2.2
**Global average**	**42.39 μM**			
**Global SD**	**4.27 μM**			
